# Imaginary Cases

**Published:** 1870-01

**Authors:** 


### IMAGINARY CASES.

CHICAGO, *December* 27, 1869.

*To the Editors of* THE CHICAGO MEDICAL JOURNAL:

I HAVE lately observed that you have begun, with a strong hand, to put down an evil that has attained no inconsiderable proportions. I mean, simply, the palming off upon the readers of professional journals of cases that only exist in the imagination of the writer, and gravely stating as facts, things that never have occurred; thus misleading many young men who naturally seek information in the pages of medical periodicals, and disgusting those whose experience enables them to see through the flimsy veil of assumed knowledge, and understand what amount of meaning there is in the *sesquipedalia* words that freely flow from their pens. Truth does not require words of such "learned length and thundering sound," but is contented to appear in the sober garb of good English, making a far better appearance than falsity does in bad Latin.

I hope you will continue to render the notoriety thus sought after less and less desirable.

In thus indorsing your cause, I know that I only express the opinion of every member of the medical profession in good standing among his fellows. So go on with the good work.

